# Primary extramedullary plasmacytoma: a rare case presentation^☆^

**DOI:** 10.1016/j.abd.2025.501137

**Published:** 2025-06-18

**Authors:** Pedro Barbosa, Clara de Diego, Javier Anaya, Corina Busso

**Affiliations:** Dermatology Service, Hospital Universitario Austral, Pilar, Buenos Aires, Argentina

Dear Editor,

Plasma cell dyscrasias are a group of clonal disorders characterized by the proliferation of neoplastic plasma cells. Plasma cell neoplasms include three categories: plasmacytoma (single lesions); plasma cell myeloma (multiple myeloma); and plasma cell neoplasms with associated paraneoplastic syndrome.^1^ Plasmacytomas develop primarily in osseous tissue (solitary plasmacytoma of bone) and less frequently in soft tissues (Solitary extramedullary plasmacytoma – SEP). The latter represent approximately 3% of neoplasms of this cell type, manifesting predominantly in the airway and gastrointestinal tract, although other organs and tissues may be involved.^2^ Cutaneous involvement is extremely rare, accounting for approximately 6% of all SEPs.^3^

We herein present a case report of an adult male who developed a primary extramedullary solitary plasmacytoma of sudden onset on his right leg.

A 66-year-old male sought consultation after the sudden onset of a lesion on his right leg. The patient denied pain or pruritus associated with the lesion. He exhibited an 8 × 8 mm painless, firm, slightly mobile erythematous-violaceous tumor, located in the right pretibial region (Fig. 1). There were no other significant cutaneous lesions or palpable lymph nodes at physical examination. The patient denied other past medical histories, did not receive any chronic medication, and did not present systemic symptoms.

**Figure 1:**
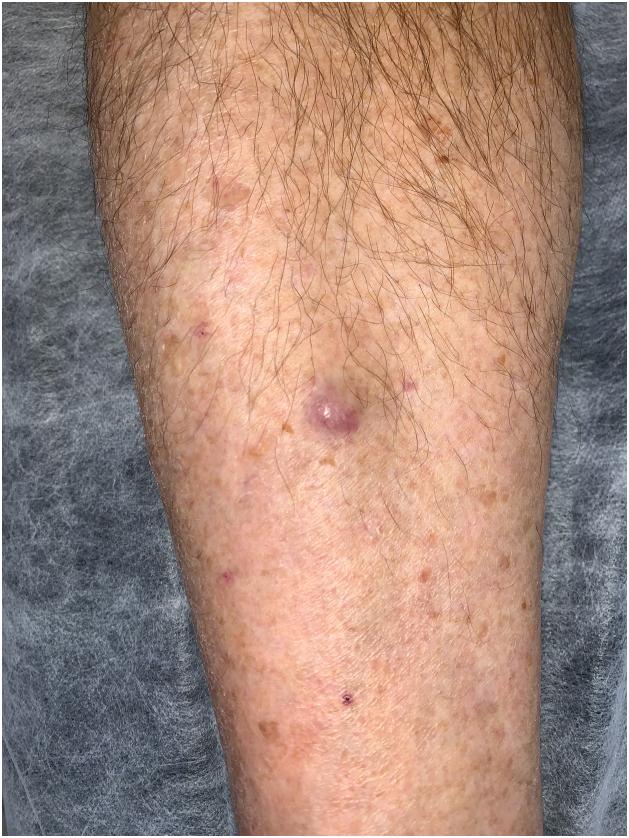
Right pretibial region; a painless, firm, slightly mobile erythematous-violaceous tumor.

A partial punch biopsy of the lesion revealed a dermal infiltration of diffuse mononuclear cells with a basophilic cytoplasm and small central nucleolus, some of which had a plasmacytoid appearance. Immunochemistry showed an atypical lymphoplasmacytic proliferation, which was CD79a positive, CD138 positive, CD20 negative, and exhibited clonality of lambda light chains (Fig. 2).

**Figure 2:**
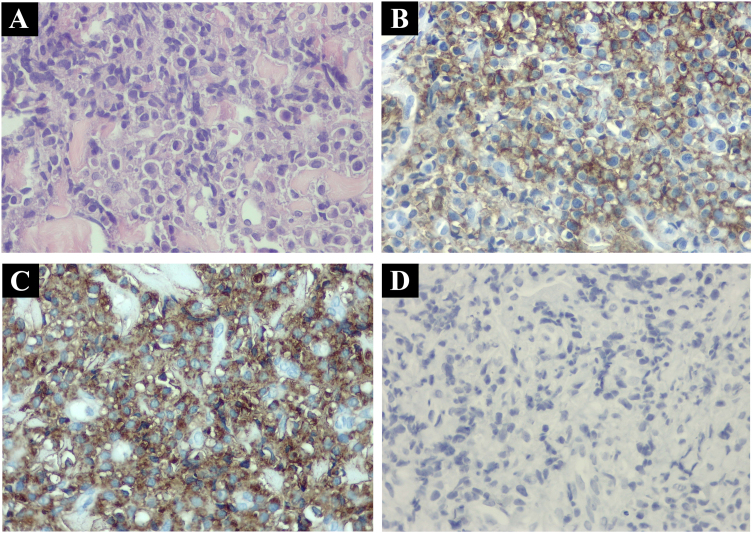
(A) Hematoxylin & eosin staining. Multiple ovoid cells with basophilic cytoplasm and eccentric nucleus demonstrating plasmacytoid differentiation. (B) CD 138positive membranous staining. (C) Lambda chain positive staining. (D) CD 20 negative staining.

An extensive workup performed in conjunction with the hematology department ruled out plasma cell myeloma. The results evidenced normal hemogram, serum electrophoretic proteinogram, and renal function; a bone marrow biopsy reported slight reactive changes but less than 5% of plasma cells, and flow cytometry showed no abnormalities. A total-body PET-scan did not reveal any hypermetabolic foci in the right leg or in other locations. These findings led us to confirm the diagnosis of solitary cutaneous extramedullary plasmacytoma.

The patient received three-dimensional conformal radiation therapy with a dose of 40 Gy over 4 weeks exhibiting a complete response. At his last follow-up a year after completion of radiotherapy, there was no evidence of cutaneous recurrence, plasma cell myeloma, or light chain disease.

Primary cutaneous plasmacytoma is a plasma cell dyscrasia (neoplasm) that is difficult to classify and diagnose due to its extreme rarity. There are fewer than 100 cases reported in the literature.^4^ The mean age of presentation is around 60, with a male predominance (approximately 3:1). The presenting single or multiple lesions are often reported as erythematous or violaceous nodules, plaques, or papules which are very rarely ulcerated and do not show any predilection site.^5^

The diagnosis of solitary extramedullary plasmacytoma requires the exclusion of associated multiple myeloma.^6^ Laboratory testing is performed to screen for evidence of end-organ damage such as anemia, hypercalcemia, or kidney impairment, and serum-free light chain ratio. Imaging such as 18F-FDG PET/CT must show no evidence of other extramedullary or lytic lesions, and a bone marrow biopsy should demonstrate no clonal plasma cells.

Therapeutic approaches for SEP include radiation, surgical excision, or a combination of both. Localized Radiation Therapy (RT) is generally the treatment of choice, most published series report using a dose of 30 to 60 Gy.^7^, ^8^ Similar studies suggest that a surgical approach with complete resection is also a viable alternative, especially in small lesions.^8^

Cutaneous SEP tends to follow an indolent clinical course, but local recurrence may occur. Dores et al. showed a 5-year overall survival of 90% for SEP presenting in skin or lymph nodes.^3^ Prognostic factors of recurrence appear to be multiple lesions and an age of onset ≥ 65.^5^, ^8^ Overall, less than 7% of patients with SEP develop local recurrences after RT, and only 10% to 15% will ultimately develop multiple myeloma.^9^ Periodic follow-up is advised every 3- to 6-months.

This case is presented due to its extreme rarity, complex clinical and histopathological diagnosis, and the necessity for strict patient follow-up, as a small percentage of patients may show local or systemic progression and develop multiple myeloma in the future.

## Authors’ contributions

Pedro Barbosa: Design and planning of the study; drafting and editing of the manuscript; collection, analysis and interpretation of data; critical review of the literature; critical review of the manuscript; approval of the final version of the manuscript.

Clara De Diego: Drafting and editing of the manuscript; collection, analysis and interpretation of data; critical review of the literature; critical review of the manuscript.

Javier Anaya: design and planning of the study, critical review of the literature; critical review of the manuscript.

Corina Busso: Design and planning of the study; effective participation in research orientation; critical review of the literature; critical review of the manuscript; approval of the final version of the manuscript.

## Financial support

None declared.

## Conflicts of interest

None declared.
